# Genetic Variation in Targets of Antidiabetic Drugs and Amyotrophic Lateral Sclerosis Risk

**DOI:** 10.3390/biomedicines12122733

**Published:** 2024-11-29

**Authors:** Mengxia Wan, Linjing Zhang, Junyan Huo, Yu Fu, Tao Huang, Dongsheng Fan

**Affiliations:** 1 Department of Neurology, Peking University Third Hospital, Beijing 100191, China; wanmengxia12@163.com (M.W.); zhanglinjing@bjmu.edu.cn (L.Z.); huojunyan1329@163.com (J.H.); lilac_fu@126.com (Y.F.); 2 Department of Neurology, Beijing Friendship Hospital, Capital Medical University, Beijing 100050, China; 3 Department of Epidemiology & Biostatistics, School of Public Health, Peking University, Beijing 100871, China; 4 Department of Global Health, School of Public Health, Peking University, Beijing 100871, China; 5 Key Laboratory of Molecular Cardiovascular Sciences, Peking University, Ministry of Education, Beijing 100871, China; 6 Beijing Key Laboratory of Biomarker and Translational Research in Neurodegenerative Diseases, Beijing 100191, China; 7 Key Laboratory for Neuroscience, National Health Commission, Ministry of Education, Peking University, Beijing 100871, China

**Keywords:** amyotrophic lateral sclerosis, antidiabetic drug, drug-target mendelian randomization

## Abstract

Background: Previous studies have suggested that antidiabetic drug use may be associated with amyotrophic lateral sclerosis. However, these studies are limited by many confounding and reverse causality biases. We aimed to determine whether antidiabetic drug use has causal effects on ALS. Methods: Drug-target Mendelian randomization analysis was conducted to evaluate the association between genetic variation in the targets of antidiabetic drugs and ALS risk. The antidiabetic drugs included sulfonylureas, GLP-1 analogues, thiazolidinediones, insulin/insulin analogues, metformin, and SGLT2 inhibitors. Summary statistics for ALS were retrieved from previous genome-wide association studies comprising 27,205 ALS patients and 55,058 controls. The instrumental variables for these drugs are from previous published articles. Results: Genetic variation in SGLT2 inhibition targets was associated with lower risk of ALS (odds ratio [OR] = 0.32, 95% CI = 0.14–0.74; *p* = 0.008). We did not find that genetic variation in metformin targets was associated with ALS (OR = 1.61, 95% CI = 0.94–2.73; *p* = 0.081). Nevertheless, mitochondrial complex I, a target of metformin, was associated with a higher risk of ALS (OR = 1.83, 95% CI = 1.01–3.32; *p* = 0.047). The analysis showed that genetic variation in sulfonylureas, GLP-1 analogues, thiazolidinediones, insulin or insulin analogues targets was not associated with ALS (all *p* > 0.05). Conclusions: The complex interaction between hypoglycemic, antioxidation, and anti-inflammatory effects may account for the different results across antidiabetic drug types. These findings provide key evidence to guide the use of antidiabetic drugs and will help to identify novel therapeutic targets in ALS.

## 1. Introduction

Amyotrophic lateral sclerosis (ALS) is an adult-onset fatal neurodegenerative disease characterized by upper and lower motor neuron degeneration and subsequent respiratory failure [1]. The etiology and pathogenesis of ALS are not fully understood, and the study of the risk factors is essential for the prevention and early intervention of ALS [2].

There is growing evidence that type 2 diabetes (T2D), one of the most common disorders of glucose metabolism, reduces the risk of ALS [3,4,5]. Case-controlled studies in Sweden, Denmark, and Italy have shown that T2D reduces the risk of ALS in older adults [3,4,5]. Mendelian randomization (MR) studies have also demonstrated that T2D reduces the risk of ALS in European populations [6,7]. Since hyperglycemia may be beneficial for ALS, hypoglycemic drugs may be harmful due to hypoglycemic lowering [8]. However, antidiabetic drugs may prevent ALS by protecting motor neurons from oxidative imbalances and glutamate-induced excitotoxicity [9,10]. With the increasing incidence of T2D at home and abroad, antidiabetic drugs are widely used as therapeutic drugs for T2D. Whether their use will increase the risk of ALS has aroused concern among researchers.

Two case-controlled studies in the Netherlands and Germany did not show an association between the use of antidiabetic drugs and the risk of ALS [11,12]. In contrast, a nested case-controlled study in Sweden reported a lower risk of ALS in the group using antidiabetic drugs, in which insulin, metformin, and sulfonylurea drugs reduced the risk of ALS [13]. A United States study also found that all antidiabetic drugs reduced the risk of ALS and glucagon was most statistically significant in reducing the risk of ALS [14]. However, observational studies could not clearly distinguish between the effects of antidiabetic drugs and T2D on ALS due to the co-existence of T2D and antidiabetic drugs. It is unclear whether individuals with longer durations of T2D appear to have lower risk of developing ALS compared with individuals with shorter durations of T2D [5].

Several experiments have explored the effects of antidiabetic drugs on ALS. Metformin has been observed to delay disease progression in a C9ORF72 genetically deficient mouse model of ALS [15]. However, in another animal study, metformin not only failed to reduce SOD1-related pathology but also increased the risk of ALS morbidity and progression in female mice in a mouse model of ALS with a SOD1 mutation [16]. Sulfonylureas and pioglitazone prolonged survival in mice with SOD1 gene deficiencies [17], but a randomized, double-blind, placebo-controlled ALS trial of riluzole plus pioglitazone was terminated early due to ineffectiveness [18].

At present, the main problems in the research of antidiabetic drugs and ALS are as follows: first, the relationship between antidiabetic drugs and ALS is not clear, and the results differ across drug types. There are many confounding factors in observational studies, which affects the accuracy of the results. Second, the correlation found by observational studies does not explain the causal association but may be due to confounding factors or reverse causal associations, and it is unclear whether antidiabetic drugs or T2D reduces ALS risk. Third, no available experimental or clinical studies have addressed the relationship between some of the newer glucose-lowering drugs and ALS, and there is a gap in the literature.

Mendelian randomization (MR) is considered a “natural” randomized controlled trial that uses genetic variation as an instrumental variable (IV) to make causal inferences between exposure and outcome, minimizing confounding influences and avoiding reverse causation [19]. MR studies can also provide information about drugs by using related gene variants to mimic drug applications, thereby predicting drug efficacy and adverse effects, which is also called drug-target MR. In previous studies, genetic related methods have shown their value in predicting adverse events and repurposing drugs because they eliminated the interference of other factors and addressed the high costs of randomized controlled trials.

**Figure 1:**
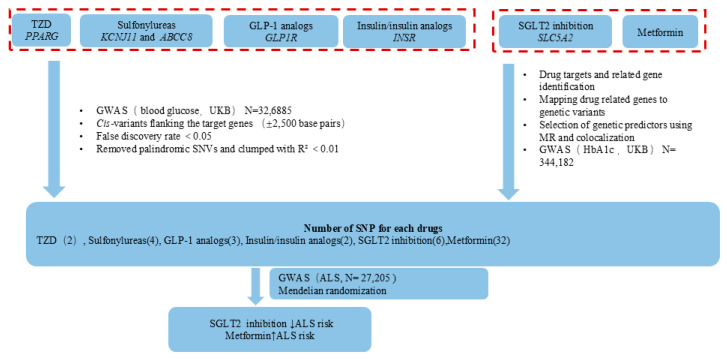
Schematic diagram of the MR assumptions underpinning an MR analysis of the association between antidiabetic drugs and ALS. MR = Mendelian randomization, ALS = amyotrophic lateral sclerosis.

Given the underexplored causal interference of antidiabetic drugs against ALS and the complex anti-inflammatory, antioxidative, and glucose-lowering roles of these antidiabetic drugs, we hypothesized that the effects of antidiabetic drugs on ALS might be different across drug categories. We performed a drug-target MR study to investigate the causal role of antidiabetic drugs in ALS. To verify this hypothesis, we conducted the most complete two-sample MR analysis based on summary statistics publicly available from large-scale GWASs with 27,205 patients with ALS in the European population. A schematic diagram of the MR assumptions underpinning an MR analysis of the association between antidiabetic drugs and ALS is shown in Figure 1.

## 2. Materials and Methods

### 2.1. Identification of Antidiabetic Drugs

We reviewed published articles and initially identified eight drugs as exposure including sulfonylureas, GLP-1 analogues, thiazolidinediones, insulin/insulin analogues, metformin, acarbose, dipeptidyl peptidase 4 (DPP-IV) inhibitors, and sodium–glucose cotransporter 2 (SGLT2) inhibitors.

### 2.2. Instrument Selection

Drug-target MR chooses mRNAs, proteins, or other downstream biomarkers as instrument of exposures to elucidate the causal association between drugs and outcomes [20]. The IVs used for the MR analysis of the drug targets remained consistent with previous studies. Unfortunately, we could not find available IV for acarbose and DPP-IV inhibitors; therefore, we performed drug-target MR for the remaining six antidiabetic drugs.

For sulfonylureas, thiazolidinediones, GLP-1 analogues, and insulin/insulin analogues, the IVs were selected from an article by Tang, B. [21], and the process is shown below. Genetic variants within the genes that encoding the protein targets of these drugs were selected in a genome-wide association study (GWAS) summary dataset for blood glucose. The genetic variants are not strongly associated (R^2^ < 0.01) [21]. The IVs were extracted from blood glucose GWAS analysis in participants of European ancestry from the United Kingdom Biobank (UKB). The UKB is a large, multicenter cohort that is one of the largest population-based studies in the world to date, with more than 500,000 participants aged 38 to 73 years [22]. This GWAS sample was for the European-ancestry population and excluded individuals with diagnoses of diabetes mellitus (defined as E10–14 in ICD 10 and 2500–2529 in ICD 9) in the inpatient registry or self-reported diabetes in the questionnaire. SNPs with minor allele frequency <0.1%, Hardy–Weinberg equilibrium *p* values < 1 × 10^−10^, and INFO scores <0.8 were excluded. In the association test, a method based on the mixed linear model was adopted, the principal components were used to control the population stratification, and the genetic relationship matrix was used to control the group kinship. Finally, 326,885 participants were analyzed which constituted the blood glucose GWAS [21]. Four types of drug targets and gene-coding information were found—sulfonylureas, GLP-1 analogues, thiazolidinediones, and insulin/insulin analogues—and the information of pharmacologically active protein targets and their corresponding coding genes were retrieved from the databases of DrugBank [23] and ChEMBL [24], respectively. The coding genes for sulfonylureas, GLP-1 analogues, thiazolidinediones, and insulin/insulin analogues are KCNJ11, ABCC8, GLP1R, PPARG, and INSR respectively. The cis-variant in each coding gene (±2,500 base pairs of gene location) was identified, and the variant associated with blood glucose was retained with a false discovery rate < 0.05. Palindromic single-nucleotide variations (SNVs), i.e., SNVs with the same pair of purine pyrimidine bases on the forward and reverse strands, were excluded to avoid confounding effect alleles. To satisfy the assumption of a strong correlation of genetic variation, the SNPs were preserved by shearing at a length of 500 kB and a level of R^2^ < 0.01. Experimentally, an additional variant, rs757110, was studied for sulfonylureas, as it has been validated as a potent sulfonylurea agent in in vitro and population studies. The instrument variables for the above four drugs were found.

Nevertheless, there are no IVs for the metformin, SGLT2 inhibitors, and DPP-IV inhibitors through the above method [21]. The IVs of the metformin and the SGLT2 inhibitors were selected from articles by Zheng, J. [25] and Xu, M. [26], and the process is shown below. The instrument variables for metformin [25] and SGLT2 inhibition [26] were identified through MR and genetic colocalization using HbA1c GWAS. The blood-glucose biomarker HbA1c-associated GWAS was derived from the European ancestry population of the United Kingdom Biobank (*n* = 344,182) [25]. For the SGLT2 inhibitors, the genetic variants associated with the mRNA expression levels of the SLC5A2 gene and the potentially functional genes of the SGLT2 inhibitors were selected using data from Genotype-Tissue Expression (GTEx) and the eQTLGen Consortium. Then, estimate the association of each SLC5A2 variant with HbA1c level and validate whether SLC5A2 and HbA1c share the same causal variant through the genetic colocalization approach. The instrument variables for metformin were selected using a similar method, except that metformin influences multiple pharmacological targets, including AMP-activated protein kinase (AMPK), mitochondrial complex 1 (MCI), mitochondrial glycerol 3 (MG3), growth differentiation factor 15 (GDF15), and glucagon-like peptide 1 (GLP1)/glucagon (GCG) [25].

### 2.3. ALS GWAS Data

We obtained large-scale European ALS GWAS summary data from a recently published study including 27,205 ALS patients and 110,881 controls, all of whom were diagnosed by professional neurologists according to the (revised) El Escorial criteria [27]. Although the patients with family histories of ALS were excluded, the ALS patients included 6374 newly genotyped cases and 22,526 control participants. This dataset contained 10,461,755 variants, and all detailed information for this study and data availability statements can be found in the original article [27].

### 2.4. Mendelian Randomization

We conducted drug-target MR in this study to estimate the causal effect of antidiabetic drugs on ALS. The MR analysis was performed under three basic assumptions: (1) an IV should be strongly associated with exposure; (2) the IV can affect outcome only through exposure instead of other direct or indirect pathways; and (3) the IV is independent of any potential confounders. We used the inverse-variance weighted (IVW) method as the main MR method. Further MR-Egger [28], weighted median, and simple median [29] tests were performed to control for horizontal pleiotropy. The Wald ratio method was used when there was only one SNP [30]. Drug-target MR analysis was also performed for each drug and separately for each pharmacological target of metformin. We also used Cochran’s Q statistic and the MR-Egger test (intercept) to test for heterogeneity and pleiotropy. If the Cochran Q test showed heterogeneity, we used the random-effect model. Otherwise, a fixed-effect model was used. If the MR-Egger intercept test showed pleiotropy (*p* < 0.05), the MR pleiotropy residual sum and outlier (MR-PRESSO) test would be used [31]. If the MR-Egger method showed pleiotropy, the results should be doubted, as it means the IV violates the second basic assumption of MR that the IV can affect outcome only through exposure.

The statistical significance level was set to 0.05 in our study.

All analysis were performed with the Two Sample MR packages (version 0.5.5) in R (Version 4.4.1).

### 2.5. Ethical Issues

All human research referred to in this study was conducted according to the Declaration of Helsinki.

## 3. Results

The genetic variants included for all the drugs and the metformin target analysis are shown separately, in Tables S1 and S2 in the Supplementary Materials. The SNPs of those IVs are not associated with ALS (*p* > 5 × 10^−5^).

**Figure 2:**
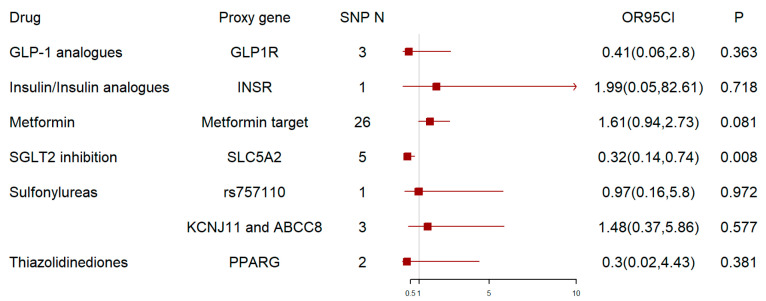
Drug-target MR analysis results. The proxy gene is the gene that encodes the drug-target protein, and we used the metformin target to refer to the gene-encoding of metformin because of its multiple pharmacological targets. rs757110 is an additional variant t which has been validated as a strong proxy in in vitro and population studies for sulfonylureas. We show the causal effect of a one-SD decrease in HbA1c via SGLT2 inhibition or metformin on ALS and the causal effect of a one-SD decrease in blood glucose via GLP-1 analogues, insulin and insulin analogues, sulfonylureas, and thiazolidinediones on ALS. GLP-1 analogues = glucagon-like peptide 1 analogues, SGLT2 = sodium–glucose cotransporter 2, SNP N = number of single-nucleotide polymorphisms, OR = odds ratio, CI = confidence interval.

This analysis showed that genetically predicted SGLT2 inhibition was associated with a lower risk of ALS (OR = 0.32, 95% CI = 0.14–0.74; *p* = 0.008) (Figure 2). In addition, the causal interference was consistent in sensitivity analysis that used weighted medians with less precision (Table S3 in Supplementary Materials). In the MR-Egger analysis, there was no evidence of directional pleiotropy (intercept 0.002 ± 0.024; *p *= 0.935) (Table 1).

**Figure 3:**
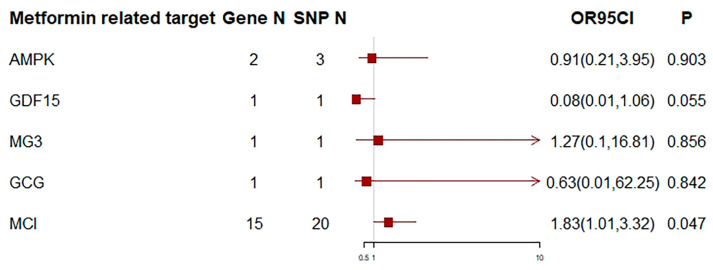
MR analysis of the effects of five metformin-related targets on ALS. We show the causal effect of a one-SD decrease in HbA1c via the five metformin-related targets. One SD unit lowering of HbA1c refers to a 6.75 mmol/mol (1.09%) reduction in the HbA1c. Gene N = number of the gene that encodes the drug target protein, SNP N = number of single-nucleotide polymorphisms, OR = odds ratio, CI = confidence interval.

Metformin may increase the risk of ALS (OR = 1.61, 95% CI = 0.94–2.73; *p* = 0.081), although that association was not statistically significant (Figure 2). To further explore the relationship between metformin and ALS, we performed an MR analysis of the five pharmacological targets, including AMPK, MCI, MG3, GDF15, and GCG, and ALS. MCI was associated with a higher risk of ALS (OR = 1.83, 95% CI = 1.01–3.32; *p* = 0.047) (Figure 3, Table S4 in Supplementary Materials). The Cochran Q test showed heterogeneity, and a random-effects model was used. The MR-Egger test did not show horizontal pleiotropy (Table 2). We did not observe a significant association between the other four targets and ALS (Figure 3).

As for another antidiabetic drugs, including GLP-1 analogues, insulin/insulin analogues, sulfonylureas, or thiazolidinediones, we did not find any effect on ALS. The results were as follows: GLP-1 analogues—OR = 0.41, 95% CI = 0.06–2.8; *p* = 0.363; insulin or insulin analogues—OR = 1.99, 95% CI = 0.05–82.61; *p* = 0.718; sulfonylureas (rs757110)—OR = 0.97, 95% CI = 0.16–5.8; *p* = 0.972; sulfonylureas (KCNJ11 and ABCC8)—OR = 1.48, 95% CI = 0.37–5.86; *p* = 0.577; and thiazolidinediones—OR = 0.3, 95% CI = 0.02–4.43; *p* = 0.381(Figure 2). We conducted sensitivity analysis to evaluate the robustness and consistency of the results, and the analysis results did not indicate heterogeneity or pleiotropy (Table 1).

## 4. Discussion

This is the first drug-target MR analysis and the most comprehensive study to evaluate the association between antidiabetic drugs and ALS. We identified the protective role of SGLT2 inhibition on ALS, and we observed that metformin may be harmful to ALS, especially through the MCI target. The complex interaction between hypoglycemic, antioxidation, and anti-inflammatory effects may lead to different results across antidiabetic types. Collectively, these findings provide key evidence to guide the use of antidiabetic drugs and help to identify novel therapeutic targets in ALS.

So far, we have not found any literature on SGLT2 inhibitors being associated with ALS. The results of this study show a causal association between SGLT2 inhibitor-related genetic variants and a reduced risk of ALS, filling a gap in the current literature. Despite the protective role of hyperglycemia toward ALS, the SGLT2 inhibitors showed a protective effect on ALS. Whether there is a pathway other than hypoglycemia that mediates the protective effect of SGLT2 inhibitors on ALS is unknown, and the protective effect of the drug in many other diseases may provide us with some ideas. In addition to hypoglycemia, SGLT2 inhibitors have many other effects, such as weight loss, lipid regulation, antihypertensive effects, lowered uric acid, improvement of non-alcoholic fatty liver disease, improvement of insulin resistance, and cardio-cerebrovascular and renal protection [32]. The mechanism may be that they may exert cardiorenal protective effects and reduce the risk of metabolic diseases through the potential off-target effects of sodium proton exchange pumps, SGLT1, ketone bodies, and erythropoietin [32]. This suggests that there may be other pathways mediating this effect.

In the nervous system, SGLT2, the target of SGLT2 inhibitors, has been shown to transport glucose in response to changes in extracellular glucose by modulating the activity of glucose transporters in neurons [33], which may play an important role in neuronal survival. Its role may be even more important when glucose levels are low or hypoxic [34]. SGLT2 inhibitors have protective effects against many neurological disorders. SGLT2 inhibitors have been shown to have potential neuroprotective effects in animal models of chronic brain diseases such as Parkinson’s disease [35]. SGLT2 inhibitors also attenuated motor dysfunction and improved motor coordination in a rat model of Parkinson’s disease-associated neurodegeneration and motor dysfunction [35]. In mouse models of epilepsy, SGLT2 inhibitors may decrease glucose consumption in neurons, thereby reducingcell membrane excitability and depolarization [36]. SGLT2 inhibitors also reduce brain damage and cognitive decline through different aspects of brain protection, such as mitochondrial function, synaptic plasticity, acetylcholinesterase activity, amyloid plaques, and modulation of the mTOR pathway [37]. SGLT2 inhibitors have many beneficial effects in humans, and we need to further explore the association and association mechanism between SGLT2 inhibitors and ALS through basic and clinical studies and explore the possibility of SGLT2 inhibitors as potential therapeutic targets for ALS.

Our study did not show a causal association between metformin and the risk of ALS (OR = 1.61, 95% CI = 0.94–2.73; *p* = 0.081), and its MCI target may increase the risk of ALS (OR = 1.83, 95% CI = 1.01–3.32; *p* = 0.047). However, metformin has been associated with a reduced risk of ALS in previous observational studies. Pfeiffer et al. [14] found that metformin reduced the risk of ALS in a case-controlled study (OR = 0.73, 95% CI = 0.73–0.93), and Mariosa et al. [13] showed similar results. In a meta-analysis, metformin reduced the risk of ALS (OR = 0.83, 95% CI = 0.75–0.93) [38]. The inverse relationship between hypoglycemic agents and the incidence of ALS in observational studies may actually be another manifestation of the neuroprotective effects of diabetes due to the confounding of diabetes [38]. However, our drug-target MR study excluded the interference of diabetes and studied the effect of metformin itself on ALS risk, which may be the reason for the inconsistency between the clinical studies and the drug-targeted MR results.

The study of metformin in the treatment of ALS also helps us understand the role of metformin. In basic experiments, metformin reduced duplication-related non-AUG proteins and improved behavior and pathology in a mouse model of ALS/frontal temporal dementia (FTD) with C9ORF72 gene mutations [15]. There is currently a clinical study evaluating the efficacy of metformin in the treatment of ALS/FTD with C9ORF72 gene mutations (ClinicalTriALS.gov registry number: NCT04220021). Paradoxically, metformin treatment did not prolong the lifespan of ALS mice in SOD1G93A mutant mouse models and may be harmful in female mice [16]. Metformin did not attenuate amyloid deposition in a yeast model [39]. ALS is a heterogeneous disease, and different types or genetic mutations of ALS may respond differently to drugs. The current research failed to distinguish ALS patients with different genetic backgrounds, which may be a reason for the failure of the clinical study. More research is needed to explore the association and possible mechanism between metformin and ALS. Physiologically, metformin has been shown to reduce hepatic glucose production, but not all effects of metformin can be explained by this mechanism. Metformin has been shown to work through AMP-activated protein kinase (AMPK)-dependent and AMPK-independent mechanisms [40]. It is also possible to inhibit mitochondrial respiration by inhibiting mitochondrial glycerol phosphate dehydrogenase, and lysosomes are also involved in the mechanisms [40]. The drug-target MR showed that the MCI target of metformin may increase the risk of ALS. The existence of other targets may weaken the harmful effect of the MCI target, and further clinical and basic studies should be carried out to explore its mechanism.

Studies have not shown the associations of sulfonylureas, thiazolidinediones, insulin and insulin analogues, and GLP1R with ALS. The results suggest that it is safe for ALS patients to use these antidiabetic drugs. The harmful effect of lowered glucose may be diluted by the protective role of anti-inflammatory agents.

There are several strengths in our study. First, the observational studies were conducted in patients with diabetes, so it remains unknown whether it is the use of a specific drug or its underlying disease that is the real relevant factor for ALS. Second, those observational studies measured drug use at baseline, but poor therapy adherence may have led to bias. Drug adherence is of less concern in drug-targeted MR models because exposure to genetic components is lifelong. Third, unmeasurable confounders can also be a problem in observational studies. Some confounding factors, which may be difficult to measure or even unknown, would result in bias. Conversely, because of the random assignment of genetic variation at the time of pregnancy, MR is expected to be less affected by confounding factors. Finally, our drug targets were analyzed positively in the relevant literature, which verified the strength of the IVs.

However, our study also has limitations. First, the results of the MR analysis represent an average linear causal effect across the general population. It is not possible to study the dose-response casual effect of drugs on ALS. Second, there are many targets of metformin, and some targets may have been missed at the current research level, resulting in a biased study. Third, due to the lifelong effect of SNPs, the effect value obtained in MR analysis is generally greater than the effect value obtained by drug RCTs, thus making it different from experimental research. Fourth, we have not yet obtained drug targets for DPP4 or α glycosidase because the mechanism of action was unclear or there was a lack of appropriate instrumental variants. Lastly, we only used genetic summary data limited to populations of European ancestry; as a consequence, the generalizability of our results is limited to European ancestral populations.

## 5. Conclusions

The complex interaction between hypoglycemic, antioxidant, and anti-inflammatory effects may account for the different results across antidiabetic drug types. These findings provide key evidence to guide the use of antidiabetic drugs and will help to identify novel therapeutic targets in ALS.

## Figures and Tables

**Table 1:** Cochran Q test results and MR-Egger intercepts in drug-target MR analysis.

Drug Class	Proxy Gene	IVW-Q Test		MR-Egger		
		Q-Statistic	Q-p	Intercept	SE	*p*-Value
GLP-1 analogues	GLP1R	0.084	0.959	−0.003	0.019	0.890
Insulin/Insulin Analogues	INSR	NA	NA	NA	NA	NA
Metformin	Metformin target	49.321	0.003	−0.003	0.008	0.755
SGLT2 Inhibition	SLC5A2	2.212	0.697	0.002	0.024	0.935
Sulfonylureas	rs757110	NA	NA	NA	NA	NA
	KCNJ11 and ABCC8	1.581	0.454	−0.012	0.026	0.719
Thiazolidinediones	PPARG	0.599	0.439	NA	NA	NA

IVW: inverse-variance weighted, SE: standard error. GLP-1 analogues = glucagon-like peptide 1 analogues, SGLT2 = sodium–glucose cotransporter 2, NA = not available.

**Table 2:** Cochran Q test results and MR-Egger intercepts in five metformin-related target MR analyses.

Metformin-Related Drug Target	IVW-Q Test		MR-Egger		
	Q-Statistic	Q-p	Intercept	SE	*p*-Value
AMPK	1.35	0.51	0	0.04	0.931
GDF15	NA	NA	NA	NA	NA
Mitochondrial-glycerol-3	NA	NA	NA	NA	NA
GCG	NA	NA	NA	NA	NA
Mitochondrial-complex-I	41.6	0.002	0.01	0.01	0.486

IVW = inverse-variance weighted, SE = standard error, NA = not available.

## Data Availability

Exposure and outcome GWASs are available in previous research, which has been shown in the Section 2 of Materials and Methods.

## Supplementary Materials

The following supporting information can be downloaded at: https://www.mdpi.com/article/10.3390/biomedicines12122733/s1, Table S1: Genetic variants used as IV in antidiabetic drug target MR; Table S2: Genetic variants used as IV in metformin drug target MR; Table S3: MR estimates of the antidiabetic drugs on ALS; Table S4: MR estimates of the each metformin target on ALS. All five targets were tested in this analysis.
